# Analysis of circRNA Differential Expression and ceRNA Network Construction in Yak Mammary Glands Across Different Physiological Stages

**DOI:** 10.3390/ani16142173

**Published:** 2026-07-13

**Authors:** Zhenyu Zhang, Yongfu La, Xiaoming Ma, Xiaoyun Wu, Min Chu, Pengjia Bao, Xian Guo, Bo Yang, Chunnian Liang

**Affiliations:** 1 Key Laboratory of Yak Breeding Engineering of Gansu Province, Lanzhou Institute of Husbandry and Pharmaceutical Sciences of Chinese Academy of Agricultural Sciences, Lanzhou 730046, China; zzy960911@163.com (Z.Z.); layongfu@caas.cn (Y.L.); maxiaoming@caas.cn (X.M.); wuxiaoyun@caas.cn (X.W.); chumin@caas.cn (M.C.); baopengjia@caas.cn (P.B.); guoxian@caas.cn (X.G.); 2 Key Laboratory of Animal Genetics and Breeding on Tibetan Plateau, Ministry of Agriculture and Rural Affairs, Lanzhou 730046, China; 3 College of Animal Science and Technology, Gansu Agricultural University, Lanzhou 730070, China; 4 Zhangye Livestock Breeding and Improvement Station of Gansu Province, Zhangye 734000, China

**Keywords:** yak, mammary gland, circular RNA (circRNA), competitive endogenous RNA (ceRNA) network, RNA-seq

## Abstract

Yak is a distinctive domestic animal adapted to the Qinghai–Tibet Plateau, and its lactation performance is restricted by extreme high-altitude conditions. Despite the growing recognition of circRNAs as important regulators of mammary gland development in other species, no study has yet systematically investigated the stage-specific circRNA expression landscapes in yak mammary glands across gestation, lactation, and non-pregnant periods, or the potential ceRNA networks through which these circRNA may participate in regulating milk synthesis and mammary cell proliferation under high-altitude conditions. This study aimed to characterize circRNA expression features and lactation regulation in yak mammary glands at the gestation, lactation, and non-pregnant stages. Total RNA sequencing was performed on mammary specimens, followed by differential screening, functional enrichment, ceRNA network construction, and qPCR verification. Numerous stage-biased circRNAs were identified, among which *circNFATC2*, *circTRPS1* and *circTAF3* were highlighted as hub candidates potentially involved in mammary cell differentiation, milk synthesis, and energy metabolism, with qPCR validating sequencing outcomes. Our results provide a comprehensive circRNA expression atlas and candidate molecular markers for understanding lactation regulation in high-altitude yaks, offering a valuable foundation for the future genetic improvement of milk production traits.

## Simple Summary

Yak milk is an important source of nutrition for herders on the Qinghai–Tibet Plateau. To improve the yield and quality of yak milk, it is necessary to elucidate the mechanisms governing mammary gland development and lactation regulation in yaks. This study collected mammary gland tissue from yaks during three physiological phases—pregnancy, lactation, and non-pregnant—to investigate changes in circRNA expression. Experimental analysis revealed that circRNA expression levels vary significantly with the mammary gland’s physiological cycle, and that certain circRNAs may be associated with the expression of genes related to mammary cell growth, milk synthesis, and energy metabolism. By constructing a molecular interaction network, key molecules regulating lactation were identified. This study systematically analyzed the dynamic expression patterns of circRNAs in yak mammary glands, suggesting potential regulatory roles of circRNAs in mammary gland development and lactation under high-altitude conditions. It provides candidate molecular markers for the genetic improvement of yak lactation performance and the enhancement of milk quality.

## 1. Introduction

The yak (*Bos grunniens*) is a ruminant endemic to the Qinghai–Tibet Plateau. It has long been adapted to the plateau’s extreme environment, characterized by low oxygen levels, low temperatures, and intense ultraviolet radiation. China has the world’s largest population of yaks and the widest distribution of the species [1]. Milk production is one of the key economic traits of yaks. Yak milk is rich in milk protein, milk fat, and functional nutrients, owing to the ecological environment of the plateau region and the grazing and semi-grazing systems. Consequently, yak milk offers advantages such as its natural, organic characteristics and high nutritional value [2]. However, due to geographical constraints and limited production capacity, yak milk yields remain low, and the development and utilization of yak milk resources are still in their early stages. Elucidating the molecular regulatory mechanisms in yak mammary tissue during different physiological stages (gestation, lactation, and non-pregnant periods) holds both theoretical significance and practical value for improving yak milk yield and quality and promoting the development of the yak milk industry.

The mammary gland is an exocrine organ that provides the nutrients and antibodies necessary for the growth of mammalian offspring and continues to undergo cycles of cell proliferation and differentiation after the newborn is weaned [3]. The mammary gland consists primarily of an extensive network of alveoli, with mammary epithelial cells tightly packed around the single-layered alveoli, all of which contribute to milk production [4]. In 1976, circular RNA (circRNA) was first discovered in viroids. circRNA is a covalently closed, non-coding RNA found in eukaryotes that exhibits tissue specificity, conservation, and stability. These characteristics make circRNA an ideal candidate for biomarkers and suggest that it may be involved in the multi-level regulation of gene expression [5]. The primary functions of circRNA include interacting with RNA-binding proteins, regulating transcription, and serving as competitive endogenous RNAs (ceRNAs). As ceRNA, circRNA contain miRNA response elements (MREs) and can competitively bind to miRNA, thereby relieving the repressive effects of miRNAs on their target mRNA and modulating gene expression at the post-transcriptional level [6]. circRNAs have been extensively studied in relation to animal growth and development, disease onset, and environmental adaptation, Li et al. [7] analyzed the circRNA expression profiles in high-IMF and low-IMF porcine longissimus dorsi muscles, identifying a total of 336 differentially expressed circRNAs, 196 circRNAs were upregulated and 140 were downregulated; dual-luciferase assays demonstrated that *circPPARA* promotes intramuscular fat accumulation in pigs by binding to *miR-429* and *miR-200b*. Wei et al. [8] analyzed the expression of circRNA in skeletal muscle tissue from embryonic and adult cattle and found that *circLMO7* inhibits myoblast differentiation and promotes cell survival via the *miR-378a-3p* pathway. circRNAs have also been studied in relation to mammalian mammary gland development and research on mammary glands diseases, Hao et al. [9] investigated the circRNA expression profiles of Xiaowei Han sheep and Gansu Alpine Merino sheep during the peak lactation period, identifying a total of 4906 circRNAs and 33 differentially expressed circRNAs. The target genes of the differentially expressed circRNA were enriched in signaling pathways related to lactation, and the ceRNA regulatory network analysis indicated that circRNA target miRNAs were associated with mammary gland development and lactation. Zhang et al. [10] performed RNA-seq on mammary tissue at 90 and 250 days postpartum, identifying 4804 and 4048 circRNAs, respectively. They found that these circRNAs contained multiple target sites for the *miR-2284* family and were predicted to target *CSN1S1* and *CSN2* mRNAs, thereby participating in the regulation of lactation-related gene expression. Zhou et al. [11] treated bovine mammary tissue with Staphylococcus aureus (10^5^ CFU/mL, MS) and PBS (control, MC) to investigate the effects of Staphylococcus aureus on circRNA expression in bovine mammary tissue. They identified seven mastitis-associated circRNA, laying a theoretical foundation for further exploration of the pathogenesis of mastitis in dairy cows. Although existing studies have established transcriptomic profiles of mammary gland development in ruminants such as dairy cows and goats, no study has yet systematically characterized circRNA expression or constructed ceRNA networks in yak mammary glands across the pregnancy, lactation, and non-pregnant stages. The yak is a unique biological model due to its adaptation to the extreme high-altitude environment of the Qinghai–Tibet Plateau, where chronic hypoxia and harsh foraging conditions impose distinct selective pressures on lactation physiology. Thus, the regulatory mechanisms inferred from low-altitude ruminants cannot be simply generalized to yaks, highlighting the need for a species-specific circRNA landscape to support genetic improvement of lactation traits in this economically important livestock.

Given the potential of circRNA to function as ceRNA in regulating mammary gland development and lactation in other mammals, we hypothesized that stage-specific circRNA in yak mammary glands may participate in similar regulatory network. To address the aforementioned research gaps, this study aims to use RNA-seq technology to analyze the circRNA expression profiles in yak mammary glands during pregnancy, lactation, and the non-pregnant period, construct a circRNA–miRNA–mRNA regulatory network, identify key regulatory nodes, and validate their expression patterns via qPCR. The findings are intended to characterize the expression patterns and identify candidate circRNA with potential regulatory roles in the yak mammary gland across different physiological stages, thereby providing a scientific basis for uncovering the molecular mechanisms governing mammary gland development and lactation regulation in high-altitude ruminants, identifying candidate molecular markers for lactation, and advancing genetic improvement in yaks.

## 2. Materials and Methods

### 2.1. Ethics Statement

All experiments involving laboratory animals were conducted in strict accordance with the Ethical Procedures and Guidelines for Animal Research in the People’s Republic of China. The experimental protocol was reviewed and approved by the Animal Management and Ethics Committee of the Lanzhou Institute of Animal Science and Veterinary Medicine, Chinese Academy of Agricultural Sciences (SYXK-2024-052).

### 2.2. Animals and Sample Collection

We selected nine healthy and disease-free adult female yaks raised at the Livestock Science and Technology Demonstration Park in Xiahe County, Gannan Tibetan Autonomous Prefecture, Gansu Province (altitude 3000 m) as test animals. The animals were 4–5 years old and weighed 130–150 kg; all were in their third lactation. The calving intervals for the first two litters of the test yaks were strictly controlled at 12 ± 0.5 months. From birth until the collection of mammary gland samples, the yaks were kept in the same rearing environment, where they had access to ad libitum feed and water and received natural lighting. The entire herd underwent uniform deworming and routine vaccination. Rearing conditions, nutritional intake, and health status were completely consistent to eliminate the interference of rearing differences on mammary gland gene expression.

The non-pregnant group consisted of randomly selected non-pregnant, non-lactating yaks (NP, *n* = 3), sampled approximately 30 days after the end of the estrous cycle; the pregnant group consisted of yaks in mid-pregnancy (approximately 200 days gestation, GP, *n* = 3), ensuring normal fetal development. The total gestation period of yaks is approximately 250–265 days. At 200 days of gestation, the mammary gland parenchyma undergoes rapid proliferation and differentiation, and a large number of alveoli are formed, marking a critical period and a key time point for the concentrated initiation and expression of genes related to lactation; The lactation group was randomly selected from the postpartum lactation peak (45 days postpartum, LP, *n* = 3). Yak reach peak lactation between 35 and 50 days postpartum; 45 days postpartum represents the time when lactation function is most vigorous and pathways related to milk component synthesis are highly activated, making it suitable for studying the transcriptomic characteristics of the mammary gland during lactation. Prepare local anesthetics and surgical instruments. Fast the yaks for 12 h and withhold water for 6 h prior to the experiment. At the posterior one-third of the mammary gland, shave the area, disinfect the surgical site with Betadine solution, and rinse thoroughly with water. Disinfect the area three times with povidone-iodine, moving from the inside out. Finally, apply medical alcohol to the surgical site. Administer local infiltration anesthesia via subcutaneous injection of 2% lidocaine hydrochloride at a dose of 0.2 mL/cm^2^. Once sensation in the surgical area has subsided, use a sterile surgical blade to make an approximately 1 cm incision through the skin and subcutaneous fascia of the right posterior mammary region, taking care to avoid major blood vessels in the mammary region. Use a scalpel and surgical forceps to obtain a 1 cm × 1 cm (approximately 1 g) tissue sample, remove any overlying tissue, transfer it to a pre-labeled cryovial, and store it in liquid nitrogen. Simultaneously, apply an anti-inflammatory agent to the incision site to prevent wound infection, and close the incision with layered sutures. Closely monitor the condition of the incision and the yak’s overall health.

### 2.3. circRNA Library Construction and Sequencing

Total RNA was extracted using the TRIzol method (Invitrogen, Carlsbad, CA, USA) according to the manufacturer’s instructions, and RNA degradation and contamination were assessed by 1% agarose gel electrophoresis. RNA purity and concentration were measured using a NanoDrop 2000 spectrophotometer (IMPLEN, Westlake Village, CA, USA), and RNA integrity was accurately assessed using an Agilent 2100 (Agilent Technologies, Santa Clara, CA, USA). After the samples pass testing, construct a cDNA library and use a specific rRNA probe to remove rRNA from the total RNA; add fragmentation buffer to randomly fragment the remaining RNA; Using RNA as a template, synthesize the first strand of cDNA with random primers; subsequently, degrade the RNA strand with RNase H, synthesize the second strand of cDNA using dNTPs as substrates, and purify the cDNA; The purified double-stranded cDNA is then subjected to end repair, A-tailing, and the addition of sequencing adapters, followed by size fractionation using AMPure XP beads; The second strand of U-containing cDNA was degraded using the USER enzyme, and the cDNA library was finally enriched via PCR. Once the library has been constructed, perform preliminary quantification using the Qubit 3.0 Fluorescence Quantifier(Thermo Fisher Scientific, Waltham, MA, USA); the concentration must be at least 1 ng/μL. Subsequently, use the Qsep 400 High-Throughput Analysis System (BiOptic Inc., New Taipei City, Taiwan) to detect the library insert fragments. Once the insert fragments meet expectations, use q-PCR to accurately quantify the effective library concentration (effective library concentration > 2 nM) to ensure library quality. Once the library construction has been verified as meeting quality standards, sequencing is performed in the PE150 format using the Illumina high-throughput sequencing platform (San Diego, CA, USA). Library quality evaluation metrics are provided in Table S1.

### 2.4. Filtering of RNA-Seq Sequencing Data from Mammary Glands Tissue

Raw RNA-seq reads were first subjected to quality control and preprocessing using fastp (v0.23.2) [12] with default parameters, including adapter trimming, removal of reads containing ambiguous bases (N), and filtering of low-quality reads (Q-score < 30). Clean reads with a minimum length of 60 bp were retained for subsequent analyses. FastQC (v0.11.9) was used to evaluate the quality of both raw and clean reads. For circRNA identification, the clean reads were aligned to the yak reference genome (*BosGru3.1*, GCA_005887515.3) using BWA-MEM (v0.7.17) [13] with default parameters. Raw read quality control and trimming were performed using the shell script 01_quality_control.sh provided in File S1.

### 2.5. Identification of circRNA

Based on circRNA structural and splicing sequence features, CIRCExplorer2 (v2.3.8) and CIRI (v2.0.6) software were used to simultaneously identify circRNAs under unified screening criteria: 1. Number of mismatches ≤ 2; 2. Number of reads spanning reverse splicing junctions ≥ 1; 3. Genomic distance between the two splicing sites < 100 kb. To improve identification accuracy, only circular RNAs detected by both algorithms were retained for subsequent analysis [14]. Statistical analyses were performed on the classification, annotation, chromosomal distribution, and length of the circRNAs. Genome alignment and circRNA identification were executed via 02_alignment.sh and 03_circRNA_identification.sh (File S1).

### 2.6. Analysis of circRNA Expression

circRNA expression levels were quantified based on back-spliced junction (BSJ) read counts and normalized to SRPBM (spliced reads per billion mapping) [15], calculated as the number of circular reads per billion total mapped reads. This normalization method corrects for variations in sequencing depth without relying on transcript length, and is therefore widely adopted for circRNA quantification as it avoids the bias introduced by length-dependent metrics such as FPKM. Only circRNAs with an SRPBM value ≥ 1 in at least two biological replicates within any physiological group were retained for downstream differential expression analysis. Differential expression analysis was performed using the edgeR package (v3.34.0) [16]. This software is based on a negative binomial distribution model and provides statistical methods for analyzing differential expression in count-based gene expression data. The obtained *p*-values were corrected using the Benjamini–Hochberg method to control the false discovery rate (FDR). circRNAs with an FDR < 0.05 and |log_2_FC| > 1 were identified as differentially expressed and retained for downstream analyses. Differential expression analysis of circRNAs was implemented in the R script 04_circRNA_analysis.R (File S1).

### 2.7. GO and KEGG Functional Enrichment Analysis of Differentially Expressed circRNA and Their Target Genes

Functional annotation of differentially expressed circRNA target genes was performed based on the GO (Gene Ontology) database across three dimensions: Molecular Function, Cellular Component, and Biological Process [17]. GO enrichment analysis was conducted using the Cluster Profiler R package(v4.2.20), and hypergeometric tests (*p* < 0.05) were employed to assess the statistical significance of enrichment for each functional term [18]. Pathway enrichment analysis was performed using the KEGG (Kyoto Encyclopedia of Genes and Genomes) database to identify significantly enriched pathways (Q-value < 0.05) [19]. The KOBAS software (v2.0) was used to assess the statistical enrichment of differentially expressed genes within KEGG pathways [20], thereby comprehensively elucidating the biological functions and pathways in which the differentially expressed genes are involved. Functional enrichment of host genes was completed with 05_enrichment_analysis.R deposited in File S1.

### 2.8. ceRNA (DEmRNA–DEmiRNA–DEcircRNA) Regulatory Network Construction

To obtain miRNA and mRNA expression data for ceRNA network construction, small RNA sequencing and mRNA sequencing were performed on the same tissue samples. For miRNA analysis, raw reads were trimmed using fastp (v0.23.2), aligned to the yak reference genome (GCA_005887515.3) using Bowtie (v1.2.3), and quantified using miRDeep2; differential expression was analyzed using DESeq2 (|log_2_FC| > 1, FDR < 0.05), with annotation based on miRBase (release 22). For mRNA analysis, clean reads were aligned using STAR (v2.7.9a), quantified with featureCounts (v2.0.1), and analyzed for differential expression using edgeR (v3.34.0) with the same thresholds.

For ceRNA network construction, circRNA–miRNA interactions were predicted using miRanda (v3.3a) with the following parameters: alignment score ≥ 140, minimum free energy (MFE) ≤ −20 kcal/mol, gap open penalty = −9, gap extend penalty = −4, and scaling parameter = 4. miRNA–mRNA interactions were predicted using both miRanda and TargetScan (release 8.0, Cow), retaining only interactions supported by both algorithms. Only circRNA–miRNA–mRNA interactions where all three molecules were differentially expressed (|log_2_FC| > 1, FDR < 0.05) were retained. The ceRNA interaction network was visualized as a Sankey diagram. The circRNA–miRNA–mRNA ceRNA regulatory network was constructed and visualized using 06_ceRNA_network.R (File S1).

### 2.9. qPCR Validation of circRNA

To validate the reliability of the transcriptomic sequencing results, this study selected eight key differentially expressed circRNA from the ceRNA regulatory network for qPCR validation. Total RNA was extracted from yak mammary tissue samples using TRIzol reagent (Takara, Dalian, China). RNA integrity and concentration were assessed via agarose gel electrophoresis and a Nanodrop spectrophotometer. Subsequently, reverse transcription was performed using a cDNA first-strand synthesis kit (TIANGEN, Beijing, China) to synthesize cDNA templates. Divergent primers were designed to target circRNA reverse splicing sites to ensure amplification of circular transcripts only and to avoid nonspecific amplification of homologous linear mRNAs. All circRNA primers and the GAPDH primer (used as an internal control) were synthesized by Shanghai Geneworks Biotechnology Co., Ltd. (Shanghai, China). Detailed sequence information is provided in Table 1. qPCR was performed using the SYBR Green SuperReal Fluorescent Quantification Kit (TIANGEN, Beijing, China) in a 20 μL reaction volume. GAPDH was used as the internal control, and the relative expression levels of each circRNA in mammary glands tissue were calculated using the 2^−ΔΔCt^ method. The results were then analyzed for consistency with the sequencing data. Primer efficiency and melting curve data for all qPCR primers are shown in Table S2 and Figure S1, respectively.

### 2.10. Statistical Analysis

All statistical analyses and graphical representations in this study were performed using IBM SPSS Statistics 26.0 and GraphPad Prism 8.0.2 (GraphPad Software Inc., San Diego, CA, USA). For comparisons among the three physiological groups (NP, GP, and LP), one-way analysis of variance (ANOVA) followed by Tukey’s honest significant difference (HSD) post hoc test was applied. For comparisons involving only two groups, an independent-samples *t*-test was used. Continuous variables are presented as mean ± standard deviation (mean ± SD). Statistical significance was set at *p* < 0.05, and all *p*-values reported for multiple comparisons were adjusted for Type I error using the Tukey correction where applicable. For RNA-seq data, differentially expressed circRNAs were identified using FDR-adjusted *p*-values (<0.05) to control for false discovery rates. Each qPCR reaction was performed in triplicate technical replicates using three biological replicates per group (*n* = 3 animals per stage), and the mean Ct values were used for subsequent analysis.

## 3. Results

### 3.1. Sequencing Data Quality and Comparison Information

This study utilized the Illumina platform for transcriptomic sequencing. After filtering out low-quality sequences and adapter sequences, 125.20 Gb of clean data was obtained, with the Q30 base percentage for each sample not falling below 88.45%. The total mapping rates of clean reads to the yak reference genome ranged from 99.88% to 99.99% across all samples, reflecting the combined contribution of uniquely and multi-mapped reads. The uniquely mapped reads accounted for 59.19% to 83.41% of the total reads, while multi-mapped reads (reads aligning to multiple genomic loci) comprised 16.57% to 40.70%. This pattern of alignment is consistent with transcriptomic analyses in yaks and other mammalian genomes with substantial repetitive sequences and gene duplications (Table 2). The high total mapping efficiency supports the reliability of downstream circRNA identification and expression quantification.

### 3.2. Overview of Identified circRNA

**Figure 1:**
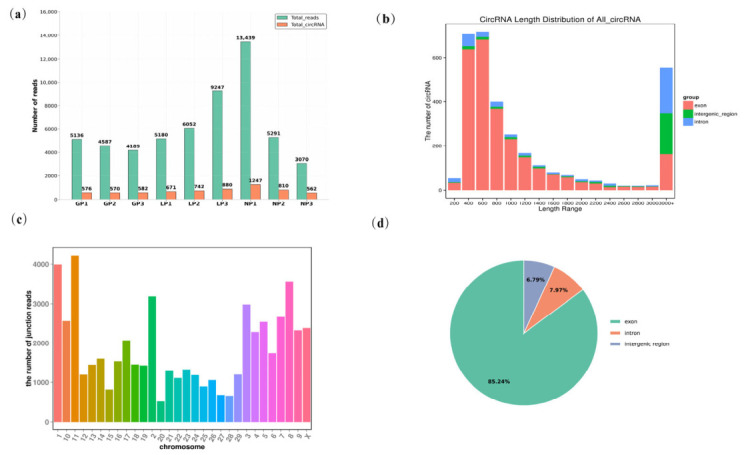
Genomic feature statistics of circRNAs identified in yak mammary glands across three physiological stages. (**a**) Statistics of total mapped reads (green bars) and number of identified circRNAs (red bars) per individual sample. The x-axis indicates sample IDs: GP (gestation period), LP (lactation period), and NP (non-pregnant period). (**b**) Length distribution of all detected circRNAs. The x-axis indicates length ranges (in nucleotides, nt), and the y-axis indicates the number of circRNAs. (**c**) Chromosomal distribution of circRNA back-spliced junction reads across yak autosomes. The x-axis indicates chromosome numbers, and the y-axis indicates the number of junction reads. (**d**) Proportional composition of circRNAs derived from exonic (85.24%), intronic (7.97%), and intergenic (6.79%) regions.

circRNA were predicted using CIRCExplorer2 (v2.3.8) and CIRI (v2.0.6) software. A total of 1728 circRNA were identified in the GP group, 2239 in the LP group, and 2619 in the NP group, laying the data foundation for subsequent differential expression analysis and functional studies (Figure 1a). In terms of circRNA length distribution, circRNA lengths were primarily concentrated in the 400–1000 nt range, consistent with the length distribution patterns of mammalian circRNA (Figure 1b); Analysis of chromosomal distribution shows that circRNA are unevenly distributed across the chromosomes of yak. Chromosomes 1, 2, 3, and 11 have relatively high numbers of circRNA junction reads, ranging from 3000 to 4000. while chromosomes 20, 27, and 28 exhibit a lower distribution of circRNA, with junction read counts typically below 700 (Figure 1c). This distribution variation may be related to differences in chromosomal gene density and functional regions, providing a distribution-level reference for subsequent studies on the genomic origins of circRNA. The results on the origin of circRNA showed that 85.24% of circRNA originated from the exonic regions of protein-coding genes, consistent with the typical characteristics of mammalian circRNA. Furthermore, the abundance of circRNA in the mammary glands of yaks at various developmental stages was comparable to that in other ruminants, such as dairy cows and sheep, suggesting that circRNA play a conserved role in regulating mammary gland development in ruminants. Only 7.97% of intron-derived circRNA and 6.79% of intergenic circRNA were identified, with exon-derived circRNA accounting for the highest proportion, suggesting that they may participate in the physiological processes of the yak mammary glandby regulating the expression of parental genes (Figure 1d). The full raw circRNA expression matrix across all samples is available in Table S3.

### 3.3. Analysis of the Expression of Differentially Expressed circRNA

**Figure 2:**
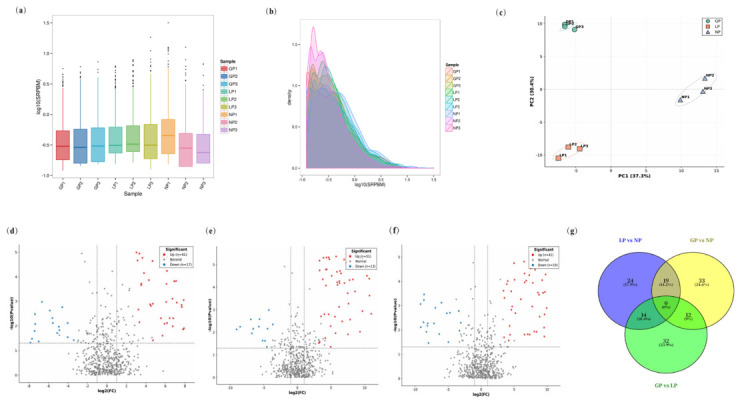
Expression profiling and differential analysis of circRNAs in yak mammary gland. (**a**) Boxplot showing circRNA SRPBM (spliced reads per billion mapping) expression distribution across samples. The x-axis indicates sample IDs (GP, LP, and NP groups), and the y-axis indicates SRPBM values (log_2_-transformed). (**b**) Density plot showing circRNA expression abundance. The x-axis indicates SRPBM values (log_2_-transformed, and the y-axis indicates density. (**c**) PCA plot for sample grouping based on circRNA expression. PC1 and PC2 represent the first and second principal components, with percentages indicating the variance explained by each component. (**d**–**f**) Volcano plots of differentially expressed circRNAs in GP vs. LP, GP vs. NP, and LP vs. NP, respectively. The x-axis indicates log_2_(fold change), and the y-axis indicates −log_10_(FDR). Red and blue dots represent significantly upregulated and downregulated circRNAs, respectively (FDR < 0.05, |log_2_FC| > 1); gray dots indicate non-significant circRNAs. (**g**) Venn diagram showing the overlap of differentially expressed circRNAs among the three pairwise comparisons. Numbers indicate the counts of circRNAs in each overlapping or unique category. No circRNAs were shared across all three comparisons.

To investigate the expression patterns of circRNAs in yak mammary glands across different physiological stages, we analyzed the SRPBM-normalized expression values of all identified circRNAs. As shown in the boxplot (Figure 2a), the expression distribution of circRNAs was generally comparable across samples, with median expression levels showing consistency within each physiological group. The density plot (Figure 2b) revealed that the majority of circRNAs exhibited relatively low expression abundance, while a small subset showed high expression levels, a pattern typical of circRNA transcriptomes. Principal component analysis (PCA) based on circRNA expression profiles clearly separated the three physiological groups (GP, LP, and NP) along the first two principal components (Figure 2c), suggesting that stage-specific circRNA expression patterns distinguish each physiological state. Differential expression analysis was performed using edgeR (v3.34.0) with screening criteria of |log2FC| > 1 and FDR < 0.05 (Benjamini–Hochberg adjusted *p*-values). Hierarchical clustering of sample expression values was conducted using Euclidean distance combined with Ward’s method to clarify the expression patterns of differentially expressed circRNAs and the consistency of sample grouping. As shown in the volcano plots (Figure 2d–f), the GP vs. LP comparison identified 58 differentially expressed circRNAs (41 upregulated and 17 downregulated); the GP vs. NP comparison identified 64 differentially expressed circRNAs (51 upregulated and 13 downregulated); and the LP vs. NP comparison identified 57 differentially expressed circRNAs (41 upregulated and 16 downregulated). The majority of differentially expressed circRNAs were upregulated, suggesting that these circRNAs may be associated with mammary gland development and lactation activation. Overlap analysis of DEcircRNAs across the three pairwise comparisons identified 134 DEcircRNAs, of which 24, 33, and 32 were uniquely expressed in LP vs. NP, GP vs. NP, and GP vs. LP, respectively. Nineteen, 14, and 12 DEcircRNAs were shared between two comparisons (Table 3), while none were common to all three (Figure 2g). These results indicate that circRNA-mediated transcriptional regulation in yak mammary glands exhibits strong physiological stage specificity, with uniquely expressed DEcircRNAs representing candidate regulators for stage-specific mammary function.

### 3.4. GO Annotation Analysis of Target Genes of Differentially Expressed circRNAs

To systematically analyze the functional characteristics of host genes of differentially expressed circRNAs in the mammary glands of yaks at different physiological stages, we performed GO functional enrichment analysis on these host genes in the GP vs. LP, GP vs. NP, and LP vs. NP groups (FDR < 0.05, fold enrichment > 1.5).

**Figure 3:**
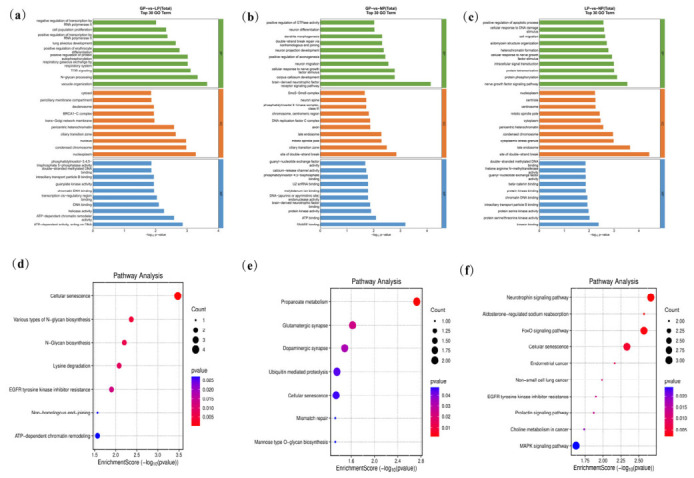
(**a**–**c**) GO enrichment analysis of DE circRNA target genes: (**a**) GP vs. LP. (**b**) GP vs. NP. (**c**) LP vs. NP. (**d**–**f**) KEGG enrichment analysis of DE circRNA target genes: (**d**) GP vs. LP. (**e**) GP vs. NP. (**f**) LP vs. NP.

In the GP vs. LP comparison, significant enrichment was observed in Biological processes terms related to vacuole organization, N-glycan processing, and TOR signaling, which may be associated with glycosylation modifications and secretory pathway establishment during lactation. Enrichment in RNA polymerase II-mediated transcriptional regulation suggests potential involvement in the transcriptional reprogramming accompanying the transition from gestation to lactation. Cellular Component analysis showed that the relevant genes were localized to the nucleus, nucleoplasm, and trans-Golgi network membrane, consistent with their possible roles in protein sorting and transport during lactation. Molecular Function analysis revealed enrichment in ATP-dependent chromatin remodeler activity, DNA binding, and phosphatidylinositol-3,4,5-trisphosphate 5-phosphatase activity, which may be linked to the PI3K-Akt-mTOR signaling axis and may contribute to milk production under high-altitude hypoxic conditions (Figure 3a).

In the GP vs. NP comparison, Biological Process terms were enriched in the brain-derived neurotrophic factor receptor signaling pathway, neuron differentiation, and dendrite morphogenesis, suggesting a potential role of neuro-mammary interactions in mammary gland development during pregnancy in yaks. Cellular Component analysis showed localization to sites of double-strand breaks and the ciliary transition zone, suggesting possible involvement of circRNA host genes in DNA damage response and cellular signaling. Molecular Function enrichment in SNARE binding, protein kinase activity, and calcium-release channel activity suggests that circRNAs may be involved in the regulation of exocytosis and calcium-dependent secretion, potentially laying a foundation for subsequent lactation. Enrichment of the phosphatidylinositol 3-kinase complex further supports the importance of the PI3K signaling pathway in mammary gland development in yaks in high-altitude regions (Figure 3b).

In the LP vs. NP comparison, Biological Process terms were enriched in the nerve growth factor signaling pathway, positive regulation of the apoptotic process, and cellular response to DNA damage stimuli, suggesting potential roles in responses to anabolic processes during lactation, oxidative stress tolerance, and maintenance of tissue homeostasis. This aligns with the adaptive regulatory characteristics of the yak mammary gland under long-term hypoxic exposure. Enrichment in cell migration and actomyosin structure organization may be associated with myoepithelial cell contraction and milk ejection. Enrichment of protein phosphorylation further suggests a role for circRNAs in lactation signaling. Cellular Component analysis showed localization to sites of double-strand breaks and late endosomes, suggesting involvement in DNA damage repair and vesicular transport. Enrichment in cytoplasmic stress granules and condensed chromosomes may reflect translation suppression and maintenance of chromosomal stability during lactation, while enrichment in centrosomes and mitotic spindles may reflect the proliferative potential of mammary epithelial cells. Molecular Function enrichment in kinesin binding, protein serine/threonine kinase activity, and beta-catenin binding suggests that circRNAs may contribute to maintaining the functional homeostasis of the lactating mammary gland by regulating cytoskeletal remodeling and cell proliferation signaling. In summary, the GO enrichment results suggest that differentially expressed circRNA host genes may precisely regulate the cyclical physiological transitions of the yak mammary gland under high-altitude conditions through the synergistic interaction of multiple modules, including metabolic adaptation, neuroregulation, stress tolerance, and lactation-specific specialization (Figure 3c).

### 3.5. KEGG Pathway Analysis of Target Genes of Differentially Expressed circRNAs

To systematically analyze the regulatory characteristics of host genes of differentially expressed circRNAs in yak mammary glands at different physiological stages, we performed KEGG pathway enrichment analysis on three comparisons: GP vs. LP, GP vs. NP, and LP vs. NP (FDR < 0.05, fold enrichment > 1.5). IIn the GP vs. LP comparison, a total of 7 significant KEGG pathways were enriched (Figure 3d). The N-glycan biosynthesis pathway was enriched, which may be associated with glycosylation processing of casein, whey protein, and other milk components during lactation. Enrichment in ATP-dependent chromatin remodeling and non-homologous end-joining pathways suggests that, during the transition from pregnancy to lactation, the yak mammary gland may adapt to high-intensity secretory activity under high-altitude hypoxia through dynamic chromatin regulation and maintenance of DNA stability. Lysine degradation was also enriched, suggesting potential involvement in amino acid metabolic reprogramming and functional remodeling of the mammary gland during the pregnancy-to-lactation transition.

In the GP vs. NP comparison, a total of 7 significant KEGG pathways were enriched (Figure 3e). Enrichment of propionate metabolism suggests a potential shift in energy substrate utilization patterns in mammary tissue from the non-pregnant to the pregnant state, possibly adapting to the energy supply requirements of a high-altitude, hypoxic environment. Enrichment of neuro-related synaptic pathways, such as glutamatergic and dopaminergic synapses, suggests a potential role of neuro-epithelial interactions during yak pregnancy in mammary gland development. Enrichment of ubiquitin-mediated proteolysis and mismatch repair pathways may contribute to protein homeostasis regulation and DNA damage repair associated with rapid mammary gland proliferation and tissue remodeling during pregnancy. Mannose-type O-glycan biosynthesis was also enriched, suggesting potential involvement in protein glycosylation during the transition from a quiescent state to pregnancy-induced development.

In the LP vs. NP comparison, a total of 14 significant KEGG pathways were enriched (Figure 3f). The prolactin signaling pathway, which is known to play a central role in the initiation and maintenance of lactation, was enriched. Enrichment of the neurotrophin, FoxO, and MAPK signaling pathways suggests that these pathways may coordinately regulate cell survival, oxidative stress response, and metabolic adaptation during lactation, potentially enhancing the stress resistance of yak mammary glands in the hypoxic plateau environment. Enrichment of the aldosterone-regulated sodium reabsorption pathway aligns with the lactating mammary gland’s need to regulate ion balance and electrolyte homeostasis in milk. Collectively, these pathways suggest that differentially expressed circRNAs may contribute to the maintenance of lactation function and physiological state transitions in yak mammary glands under high-altitude hypoxic conditions by regulating pathways related to lactation signaling, energy metabolism, stress tolerance, and ion balance.

### 3.6. Construction of ceRNA (DEmRNAs-DEmiRNAs-DEcircRNA) Regulatory Networks

**Figure 4:**
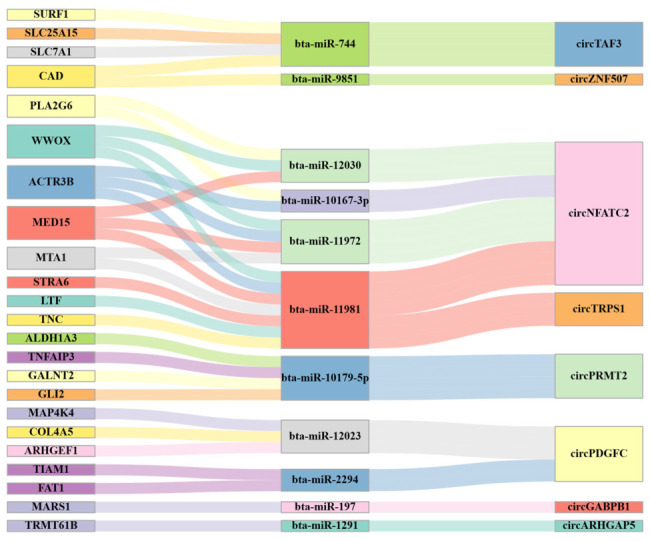
Sankey diagram of the core circRNA–miRNA–mRNA ceRNA regulatory network in yak mammary gland. Right, core hub circRNA; middle, targeted miRNAs; left, downstream target mRNAs.

To systematically analyze the circRNA mediated competitive endogenous RNA (ceRNA) regulatory patterns in the yak mammary gland during different physiological phases (GP, LP, NP), this study constructed an integrated circRNA–miRNA–mRNA ceRNA regulatory network based on differentially expressed circRNAs, miRNAs, and mRNAs. This network comprises 8 core differentially expressed circRNAs (DEcircRNA) nodes, 11 DEmiRNAs nodes, and 23 DEmRNAs nodes, exhibiting a typical cascade regulatory topology, and provides insights into the molecular mechanisms underlying physiological transitions in the yak mammary gland under high-altitude conditions (Figure 4).

*circNFATC2*, *circTRPS1*, and *circTAF3* are key hub molecules in the network; *circNFATC2* competitively binding to *bta-miR-11981*, *bta-miR-11972*, *bta-miR-12030*, and *bta-miR-10167-3p*, to regulate downstream genes *MED15*, *MTA1*, *ACTR3B*, *WWOX,* and *PLA2G6*, thereby participating in mammary epithelial differentiation, transcriptional regulation, and lipid metabolism; *circTRPS1* regulates *STRA6*, *LTF*, and *TNC* by sponging *bta-miR-11981*, linking mammary gland development, immune defense, and extracellular matrix remodeling, thereby enhancing the mammary gland’s disease resistance and tissue remodeling capacity in high-altitude environments; *circTAF3* binds to *bta-miR-744* to regulate *SURF1*, *SLC7A1*, *CAD*, and *SLC25A15*, participating in mitochondrial energy metabolism, amino acid transport, and nucleotide synthesis, thereby meeting the high energy and anabolic demands of lactation under hypoxic conditions. Furthermore, *circPDGFC*, *circPRMT2*, *circZNF507*, *circGABPB1*, and *circARHGAP5* also participate in network regulation. Through *bta-miR-12023*, *bta-miR-2294*, *bta-miR-10179-5p*, *bta-miR-9851*, *bta-miR-197*, and *bta-miR-1291* to regulate target genes such as *TIAM1*, *COL4A5*, *ALDH1A3*, and *MARS1*, which are involved in biological processes including cytoskeletal remodeling, milk protein synthesis, and oxidative stress homeostasis, respectively. This network, constructed based on high-throughput sequencing and bioinformatics predictions, provides reliable candidate molecules and regulatory clues for the regulation of lactation in yaks. The aforementioned core circRNAs can serve as priority candidate molecules for subsequent studies on yak lactation traits, providing theoretical support for elucidating the mechanisms of mammary gland development in high-altitude ruminants and for molecular breeding.

### 3.7. qPCR Validation

**Figure 5:**
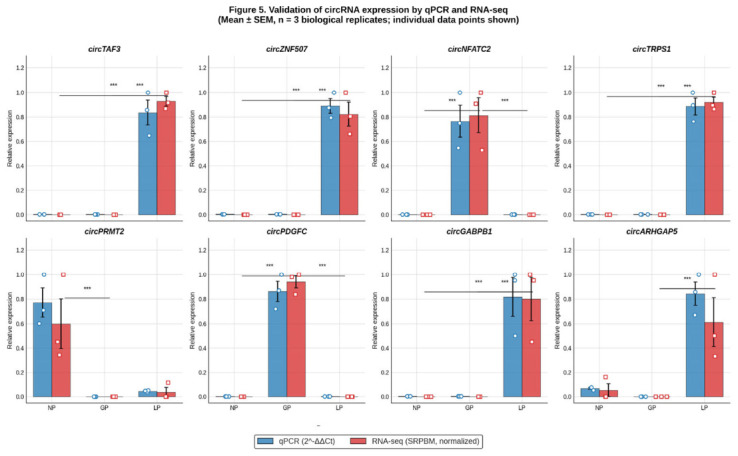
Validation of circRNA expression by qPCR and RNA-seq. Relative expression of eight circRNAs in yak mammary glands across gestation (GP), lactation (LP), and non-pregnant (NP). qPCR data (bars, left y-axis) were normalized to GAPDH and calculated using the 2^−ΔΔCt^ method (mean ± SEM; *n* = 3 biological replicates; individual data points shown). RNA-seq data (lines, right y-axis) are shown as SRPBM-normalized expression values (mean ± SEM). Statistical significance was determined by one-way ANOVA followed by Tukey’s HSD post hoc test (*** *p* < 0.001; ns, not significant). Error bars represent SEM. The concordance between qPCR and RNA-seq expression trends validates the reliability of the transcriptomic data.The 2 × 4 layout is arranged as Row 1: *circTAF3*, *circZNF507*, *circNFATC2*, *circTRPS1*; Row 2: *circPRMT2*, *circPDGFC*, *circGABPB1* and *circARHGAP5*.

To validate the reliability of the RNA-seq results, this study selected eight key differentially expressed circRNAs from the ceRNA regulatory network for qPCR validation. The results showed that the expression trends of these 8 core circRNAs were consistent with those observed in the RNA-seq analysis, confirming the accuracy and reliability of the sequencing data (Figure 5). Furthermore, the candidate circRNAs exhibited stage-specific expression patterns, suggesting that they play important regulatory roles in the cyclical transition of the yak mammary gland. Specifically, *circNFATC2* and *circPDGFC* were specifically upregulated during pregnancy; *circTAF3*, *circZNF507*, *circTRPS1*, *circGABPB1*, and *circARHGAP5* exhibited the highest expression levels during lactation; and *circPRMT2* was highly expressed during the non-pregnant phase. These stage-specific expression patterns further indicate that circRNAs may participate in the dynamic regulatory processes of mammary gland development during pregnancy, functional maintenance during lactation, and remodeling during the non-pregnant phase in yak, providing reliable expression validation support for the screening of key circRNAs in this study.

## 4. Discussion

### 4.1. Genomic Features of Yak Mammary circRNAs

This study systematically characterized the dynamic circRNA expression profiles in mammary tissue from yaks during the gestation period (GP), lactation (LP), and non-pregnant (NP) periods. A total of 125.20 Gb of high-quality RNA-seq data was obtained, with 1728–2619 circRNAs identified in each group. Differential expression analysis revealed 58, 64, and 60 differentially expressed circRNAs in the GP vs. LP, GP vs. NP, and LP vs. NP comparisons, respectively. GO and KEGG enrichment analyses indicated that the host genes of differentially expressed circRNAs are primarily involved in RNA metabolism, cell differentiation, energy metabolism, neural signaling, and the prolactin signaling pathway. A predicted ceRNA network comprising 8 circRNAs, 11 miRNAs, and 23 mRNAs was further constructed, in which *circNFATC2*, *circTRPS1*, and *circTAF3* were identified as hub nodes with potential involvement in mammary differentiation, lipid metabolism, energy homeostasis, and immune defense through predicted competitive binding of miRNAs. In summary, this study provides the first comprehensive resource of circRNA expression profiles and a predicted ceRNA regulatory network during the physiological transition of the yak mammary gland.

High-throughput sequencing data (Q 30 ≥ 88.45%, coverage 99.88–99.99%) support the reliability of subsequent analyses. The number of circRNAs identified in yak mammary glands (1728–2619) is comparable to that reported in sheep mammary glands (2369 circRNAs) [21] and partially overlaps with the range observed in dairy cows (4804 [22] and 4048 [23] at different time points). These comparisons suggest that the abundance of circRNAs in mammary tissue may be broadly comparable across ruminant species, potentially reflecting conserved regulatory features; however, this inference is indirect and requires functional validation. The lengths of circRNAs are primarily concentrated in the 400–1000 nt [24], which is consistent with general trends, as circRNAs that are too long or too short tend to be less stable or difficult to assemble [25]. Chromosomes 1, 2, 3, and 11 exhibited relatively high numbers of back-splicing reads. This distribution is consistent with previous observations that circRNA abundance is positively correlated with gene density and transcriptional activity in several mammalian genomes [26,27]. However, the relationship between chromosomal location and circRNA biogenesis remains incompletely understood, and the higher circRNA abundance observed on these chromosomes may reflect, at least in part, the genomic features of these regions.

### 4.2. Functional Enrichment of Differentially Expressed circRNAs

GO enrichment analysis revealed significant enrichment in categories such as vacuolar organization, N-glycan processing, and TOR signaling. These enriched categories may be associated with the establishment of secretory pathways [28] and mTOR-mediated milk protein synthesis [29]; however, this inference is based on pathway enrichment rather than direct experimental assessment of mTOR activity, and should therefore be considered suggestive rather than confirmatory. These findings provide candidate clues for understanding how circRNAs might participate in lactation initiation. The enrichment of RNA polymerase II-mediated transcriptional regulation suggests a potential link between the host genes of circRNAs and the transcriptional reprogramming that accompanies lactation onset [30]. Cellular localization analysis showed that the relevant genes are enriched in the nucleus and the trans-Golgi network membrane, which is consistent with their possible involvement in the sorting of milk components such as milk proteins and lactose [31] and in secretory transport [32]. KEGG pathway enrichment analysis revealed significant enrichment in pathways such as N-glycan biosynthesis and ATP-dependent chromatin remodeling. These pathways are suggestive of metabolic and regulatory adaptations in yak mammary glands during the lactation initiation phase, particularly in protein processing [33], chromatin dynamics [34], and signal transduction [35], which may collectively contribute to the rapid activation of lactation under high-altitude conditions. Comparative analysis of the pregnant and non-pregnant states (GP vs. NP) highlights pathways related to neuroregulation, including brain-derived neurotrophic factor (BDNF) receptor signaling, neuronal differentiation, and dendritic morphogenesis. The mammary gland is co-regulated by the nervous and endocrine systems [4], and neurotrophic factor signaling, such as BDNF-TrkB, is involved in mammary gland morphogenesis and the maintenance of stem cell homeostasis [36]. At the molecular functional level, SNARE proteins are significantly enriched in calcium release channels, suggesting that circRNAs may be associated with the regulation of exocytosis [37] and calcium-dependent secretion [38], potentially laying the groundwork for the efficient release of lactation-related components. KEGG analysis revealed enrichment of pathways such as propionate metabolism and mannose-type O-glycan biosynthesis, suggesting that when yak mammary glands transition from a non-pregnant quiescent state to a proliferative state during pregnancy, this is accompanied by systemic metabolic reprogramming involving energy substrate utilization [39] to meet the demands of tissue development in the hypoxic environment of the plateau. In the comparison between the lactation period and the non-pregnant period (LP vs. NP), the prolactin signaling pathway was significantly enriched, suggesting that this pathway may play a key role in the initiation and maintenance of lactation in yaks, and suggesting that circRNA host genes may be involved in the regulation of the lactation signaling pathway. Enrichment of neurotrophic factor, FoxO, and MAPK signaling pathways suggests that circRNAs may be associated with cell survival, oxidative stress response, and metabolic homeostasis regulation during the high-intensity anabolic processes of lactation [40], potentially enhancing the mammary gland’s adaptability and stress resistance in high-altitude environments. In addition, the enrichment of DNA damage response and apoptosis regulatory pathways may reflect the physiological stress associated with sustained high-intensity synthetic activity in the lactating mammary gland [41], and may suggest that related circRNAs could be involved in the repair of stress-induced damage and the maintenance of tissue homeostasis [42].

### 4.3. ceRNA Regulation and qPCR Validation of Hub circRNAs

The ceRNA network constructed in this study provides a preliminary framework for understanding how circRNAs might participate in lactation regulation through the miRNA sponge mechanism. *circNFATC2*, *circTRPS1*, and *circTAF3* were identified as core hub nodes within this network and represent promising candidates for future functional studies on their potential roles in yak mammary gland development and lactation. *circNFATC2* was predicted to specifically bind to *bta-miR-11981*, *bta-miR-11972*, *bta-miR-12030*, and *bta-miR-10167-3p*, thereby regulating the downstream target genes *MED15*, *MTA1*, *ACTR3B*, *WWOX*, and *PLA2G6* [43]. *MED15* is a component of the Mediator complex that integrates transcription factor signals and regulates lipid metabolism [44]; *MTA1* is associated with chromatin remodeling and mammary glands development [45]; *PLA2G6* encodes a phospholipase involved in phospholipid remodeling and arachidonic acid release [46], which influences the efficiency of milk fat synthesis. Therefore propose the following hypothesis: *circNFATC2* may be involved in the synthesis of milk fat and the maintenance of lactation in yaks by coordinating processes related to transcriptional regulation, chromatin remodeling, and lipid metabolism—a function that, if confirmed, would be consistent with the high-fat milk characteristic of yaks. However, we emphasize that this remains a hypothesis derived from bioinformatic predictions and requires experimental validation. *circTRPS1* was predicted to specifically bind to *bta-miR-11981* to regulate *STRA6*, *LTF*, and *TNC*. *LTF* is an important antimicrobial protein in the mammary gland and a signature molecule of the lactation phase [47]; *STRA6* participates in retinol (vitamin A) signaling [48] and regulates the proliferation and differentiation of mammary epithelial cells; *TNC* (tenascin C) is an extracellular matrix protein involved in tissue remodeling [49]. Therefore, *circTRPS1* may link immune defense, vitamin A metabolism, and tissue remodeling processes, thereby enhancing the disease resistance and adaptability of yak mammary glands in high-altitude environments. *circTAF3* was predicted to specifically bind to *bta-miR-744* to regulate genes involved in energy metabolism and substance transport, such as *SURF1*, *SLC7A1*, *CAD*, and *SLC25A15*. *SURF1* is involved in the assembly of mitochondrial respiratory chain complex IV (cytochrome c oxidase) [50], supporting the high energy demands of lactation; *SLC7A1* (cationic amino acid transporter) and *SLC25A15* (mitochondrial ornithine transporter) are involved in amino acid uptake [51] and mitochondrial metabolism [52]; *CAD* (carbamoyl phosphate synthetase 2, aspartate carbamoyltransferase, and dihydroorotate synthase) is a key rate-limiting enzyme in pyrimidine synthesis [53], therefore, *circTAF3* may meet the high-intensity anabolic demands required for lactation maintenance in the hypoxic environment of high-altitude regions by regulating energy metabolism, amino acid transport, and nucleotide synthesis. Other circRNAs (*circPDGFC*, *circPRMT2*, *circZNF507*, *circGABPB1*, *circARHGAP5*) also participate in cytoskeletal remodeling, milk component synthesis, and the regulation of oxidative stress homeostasis through their corresponding miRNAs. These molecules form a multi-level regulatory network that may collaboratively participate in functional transitions and lactation regulation during different physiological stages of the yak mammary gland at the levels of substance synthesis, energy metabolism, immune defense, and tissue remodeling, providing candidate molecules and regulatory clues for further investigation of lactation mechanisms in high-altitude ruminants. Based on high-throughput sequencing and qPCR-based expression validation, this study systematically characterized the expression profiles of circRNAs and predicted ceRNA regulatory networks in the mammary glands of yaks at different physiological stages. While qPCR validation supported the reliability of the expression patterns, we acknowledge that it does not validate the proposed ceRNA interactions; functional experiments (such as Luciferase reporter assays, RNA pull-down) are required to confirm the predicted circRNA–miRNA–mRNA regulatory relationships.

### 4.4. Limitations and Future Perspectives

This study focused solely on bioinformatics predictions and quantitative validation of gene expression; molecular interaction assays, such as dual-luciferase assays and RNA pull-down experiments, have not yet been conducted. Due to the constraints of the high-altitude environment, obtaining fresh mammary tissue samples from live yaks via surgical biopsy is technically challenging and costly. Consequently, the sample size per group is limited, which may result in incomplete detection of some low-abundance circRNAs. However, the experimental protocol followed standard procedures, and the expression trends of core circRNAs were validated via qPCR. The resulting regulatory network and key candidate molecules provide insights into the stage-specific regulatory patterns in yak mammary glands, offering valuable targets for future studies. Future research could expand the sample population and utilize in vitro mammary epithelial cell culture models to conduct overexpression and knockout functional assays targeting key genes such as *circNFATC2*, *circTRPS1*, and *circTAF3*; utilize dual-luciferase assays to precisely validate circRNA–miRNA binding interactions; and, in conjunction with cellular phenotypic assays, investigate the regulatory roles of key circRNAs in mammary cell proliferation and the synthesis of milk fat and proteins, thereby refining the understanding of lactation regulation mechanisms in high-altitude yaks at the cellular and molecular levels.

## 5. Conclusions

In this study, RNA-seq was performed on mammary gland tissues from yaks during pregnancy, lactation, and the non-pregnant period to systematically investigate the expression patterns and regulatory functions of circRNAs across different physiological stages. A total of 1728, 2239, and 2619 circRNAs were identified in the three groups, respectively, with the majority originating from exonic regions and ranging in length from 400 to 1000 nt. Pairwise comparisons between groups identified differentially expressed circRNAs, respectively. Functional enrichment analysis revealed that the target genes of differentially expressed circRNAs were enriched in RNA metabolism, cell differentiation, energy metabolism, neural signaling, and prolactin-related pathways closely associated with mammary gland development and lactation. Concurrently, a ceRNA regulatory network comprising 8 circRNAs, 11 miRNAs, and 23 mRNAs was constructed, in which circNFATC2, circTRPS1, and circTAF3 were identified as hub nodes. These circRNAs were predicted to participate in mammary epithelial differentiation, lipid synthesis, energy metabolism, and immune homeostasis through competitive binding to target miRNAs. qPCR validated the expression patterns of the 8 circRNAs, confirming that their distinct stage-specific expression profiles were consistent with the sequencing results. Importantly, while these findings identify candidate circRNAs associated with mammary gland development, we acknowledge that the proposed ceRNA interactions and regulatory functions are predicted based on bioinformatic analyses and require experimental validation (e.g., Luciferase reporter assays, RNA pull-down, or functional knockdown/overexpression studies) to confirm their biological relevance. In summary, this study characterized the circRNA expression profiles and predicted ceRNA regulatory networks in the cyclic development of yak mammary glands under high-altitude plateau conditions, providing a basis for identifying for future genetic improvement and functional studies in yaks.

## Figures and Tables

**Table 1:** Primers used for qPCR validation of candidate circRNA.

circRNA ID	Forward (5′ → 3′) Reverse (5′ → 3′)	Product Length (bp)
*circTAF3*	F: GGATCCACTTTGATTTTCTCATG R: CCTGTGGGTACCTGCTCTTCTTCT	135
*circZNF507*	F: GGAAGAAGGCAGCAGTGTTGCC R: TGTGTCACCAAACCTCAGAAAG	178
*circNFATC2*	F: CCGAGTGCACATAAGGTGGCCA R: TCCATCTTGCTGGTGCCACCCT	192
*circTRPS1*	F: ACTTCAGGTGGAACATTCATTGGC R: TCCGGCCCCCAGGAGCAGACGA	210
*circPRMT2*	F: TTCACTTGGAGATGTTGGCAGA R: GCAGCACCACGTCTTCCACC	150
*circPDGFC*	F: GAGGAACTATACCCAAGCATCT R: GTGCTCCCAAGAGCAGTTCT	190
*circGABPB1*	F: TTTAGATATATTCAGTGGGACCT R: GTTCGGAAGATTGGACAGTGGA	185
*circARHGAP5*	F: AAGATGATCCATATGATCTTGAAGAC R: GTATTTTGGGATGCCCCTCCAG	188
*GAPDH*	F: CTGACCTGCCGCCTGGAG R: GTAGAAGAGTGAGATGTCGCTGTTG	149

**Table 2:** Summary of sequencing data and mapping statistics.

Sample ID	Total Reads	Mapped Reads	Uniq Map Reads	Multiple Map Reads	Q30 (%)
GP1	111,269,756	111,236,664 (99.97%)	88,150,113 (79.22%)	23,086,551 (20.75%)	92.65
GP2	93,362,628	93,322,592 (99.96%)	73,849,108 (79.10%)	19,473,484 (20.86%)	94.05
GP3	88,350,798	88,330,698 (99.98%)	71,944,971 (81.43%)	16,385,727 (18.55%)	94.65
LP1	86,030,654	86,018,418 (99.99%)	71,760,015 (83.41%)	14,258,403 (16.57%)	93.92
LP2	81,548,226	81,521,990 (99.97%)	66,842,317 (81.97%)	14,679,673 (18.00%)	94.30
LP3	106,705,542	106,669,036 (99.97%)	84,957,997 (79.62%)	21,711,039 (20.35%)	95.24
NP1	89,728,268	89,691,156 (99.96%)	66,968,772 (74.64%)	22,722,384 (25.32%)	94.18
NP2	96,920,578	96,811,904 (99.89%)	58,492,318 (60.35%)	38,319,586 (39.54%)	90.13
NP3	86,103,642	86,002,718 (99.88%)	50,962,450 (59.19%)	35,040,268 (40.70%)	88.45

**Table 3:** Summary of differentially expressed circRNA among three physiological groups of yak mammary gland.

Group	DEG Number	Up-Regulated	Down-Regulated
GP vs. LP	58	41	17
GP vs. NP	64	51	13
LP vs. NP	60	41	19

## Data Availability

The raw RNA-seq data generated in this study have been deposited in the NCBI Sequence Read Archive (SRA) under BioProject accession number PRJNA1477671 (https://www.ncbi.nlm.nih.gov/sra/PRJNA1477671, accessed on 8 July 2026). The data will be publicly available upon publication.

## Supplementary Materials

The following supporting information can be downloaded at: https://www.mdpi.com/article/10.3390/ani16142173/s1, Table S1. Raw circRNA count matrix. Table S2. Library strandedness evaluation (RSeQC). Table S3. Primer efficiencies and Tm. Figure S1. Melting curves. File S1. Analysis scripts.
