# Supplementary material for: Analysis of circRNA Differential Expression and ceRNA Network Construction in Yak Mammary Glands Across Different Physiological Stages
Source: Animals (Basel). 2026 Jul 13;16(14):2173. doi: 10.3390/ani16142173 (PMC13405935; doi:10.3390/ani16142173)
Supplement: Supplementary file 1 [file animals-16-02173-s001.zip › Table S1-Raw circRNA count matrix.pdf]

#sample_id	read1_vs_gene	read2_vs_gene	undefined
GP1	0.01	0.99	0
GP2	0.011	0.99	0
GP3	0.012	0.99	0
LP1	0.0093	0.99	0
LP2	0.011	0.99	0
LP3	0.013	0.99	0
NP1	0.025	0.97	0
NP2	0.03	0.97	0
NP3	0.025	0.97	0

The table shows the proportion of reads aligned to the sense or antisense strand of annotated genes. **read1_vs_gene** indicates the fraction of read1 aligned consistently with the gene strand; **read2_vs_gene** indicates the corresponding fraction for read2; undefined represents reads mapping to regions with bidirectional transcription where strand assignment could not be determined. **The data confirm that the libraries are stranded (fr-secondstrand type), with read2 consistently mapping to the sense strand (> 97%) and read1 to the antisense strand across all samples**.
