# Supplementary figures and images for: Analysis of circRNA Differential Expression and ceRNA Network Construction in Yak Mammary Glands Across Different Physiological Stages
Source: Animals (Basel). 2026 Jul 13;16(14):2173. doi: 10.3390/ani16142173 (PMC13405935; doi:10.3390/ani16142173)

Melt Curve

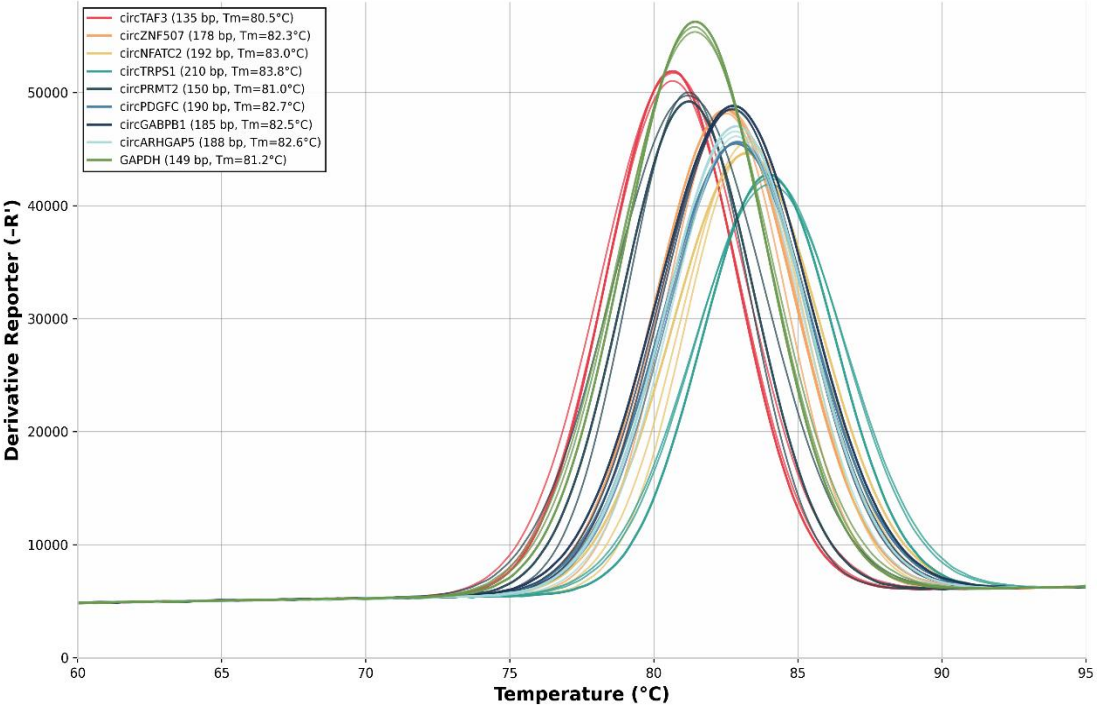

Supplement: Supplementary file 1 [file animals-16-02173-s001.zip › Figure S1-Melting curves.pdf]
